# Which is the Superior Thoracolumbar Injury Classification Tool? TLICS Versus AOSpine 2013: A Systematic Review

**DOI:** 10.1177/21925682241311303

**Published:** 2024-12-25

**Authors:** Kristina T. Pidd, David Sadauskas, Vanesa Tomatis, Ema J. Knight

**Affiliations:** 1 School of Medicine, 1065 Flinders University, Adelaide, SA, Australia; 2 Department of Neurosurgery, 14351 Flinders Medical Centre, Adelaide, SA, Australia

**Keywords:** thoracolumbar spine, AOSpine, TL AOSIS, TLICS, spinal trauma, conservative management, spine injury classification

## Abstract

**Study Design:**

Systematic Literature Review.

**Objectives:**

To address whether TLICS or AOSpine is best used in clinical practice through assessment of interobserver and intraobserver reliability, agreement, and imaging modality performance.

**Methods:**

This systematic literature review was reported in accordance with PRISMA 2020 guidelines. Articles were included based on meeting eligibility criteria: studies evaluating TLICS, AOSpine, and/or TL AOSIS through reliability, agreement, or imaging modality performance with adult patients (≥18) suffering from traumatic thoracolumbar fractures. Articles were acquired in April 2023 from Medline, CINAHL, and Scopus. Risk of bias was assessed through a modified COSMIN checklist. Tabulated results were separated by classification tool (TLICS or AOSpine/TL AOSIS) and reliability, agreement, or imaging modality results.

**Results:**

Twenty-one studies were included in the final review. Interobserver and intraobserver AOSpine morphology reliability was on average superior to TLICS. Increased familiarity with the tool positively influenced both AOSpine and TLICS performance. For surgical treatment recommendation, AOSpine differentiated between stable and unstable burst fractures and guided clinician’s more accurately than TLICS. Regarding conservative treatment, both TLICS and AOSpine reported similar clinical accuracy. TLICS performed significantly better when MRI was incorporated compared to CT alone. CT was sufficient as an imaging modality for AOSpine/TL AOSIS performance.

**Conclusions:**

AOSpine outperformed TLICS in surgical reliability, agreement and did not require additional MRI imaging to improve accuracy. Limitations of evidence include low quality of available studies and significant heterogeneity in patient and observer number. Future prospective multicentre research is recommended. This study was not funded and not registered on PROSPERO.

## Introduction

Traumatic fractures of the thoracolumbar spine (TL) are treated surgically or conservatively, with decision making aided in part by classification systems. Despite efforts to create a universally accepted method of classification, there is still ongoing debate about which system is best for clinical practice. Since the inception of the first thoracolumbar classification system by Bohler^1^ there have been numerous attempts to improve reliability and clinical agreement. Of the systems that have been published, the Thoracolumbar Injury Classification and Severity Score (TLICS) and the AOSpine Thoracolumbar Spine Injury Classification System (AOSpine) are the most modern and thus the focus of this paper.^2,3^

Proposed in 2005, TLICS was designed to improve upon the thoracolumbar injury severity score (TLISS).^4^ TLICS is composed of three variables: fracture morphology, integrity of the posterior ligamentous complex (PLC), and neurological status.^3^ Points are assigned to each subcomponent, yielding a numerical score to guide treatment approach: 0-3 = non-operative, 4 = operative or non-operative, and ≥5 = operative.^5^

AOSpine was introduced in 2013 as a hybrid of the AO/Magerl and TLICS systems.^2^ Designed to be simple and reproducible, it uses three categories: fracture morphology, neurological status, and patient-specific modifiers.^2^ The Thoracolumbar AOSpine Injury Score (TL AOSIS) was developed in 2016 to accompany AOSpine.^6^ Scores are interpreted as 0-3 = non-operative, 4-5 = operative or non-operative, ≥6 = operative.

An ideal classification system should meet the following criteria: be reliable, be clinically useful (direct general guidelines of treatment accurately), and effectively incorporate knowledge of the biomechanics of thoracolumbar injuries with modern imaging modalities (CT, MRI).^7^ The necessity of MRI is polarising,^8-10^ and therefore is of interest to explore how this imaging modality influences the performance of TLICS and AOSpine/TL AOSIS.

As there is still contention in the literature, the aim of this systematic review is to answer two questions: “Is TLICS or AOSpine/TL AOSIS superior in reliability and agreement?” and “How does imaging modality influence TLICS and AOSpine classification and score?” to ultimately address which tool is best used in clinical practice. This was achieved by investigating three components:

1. Interobserver and intraobserver reliability of TLICS and AOSpine
2. Agreement between TLICS or TL AOSIS and clinician treatment
3. Classification and score change based on CT +/− MRI (imaging modality influence as a percentage)

Due to the potentially misleading nature of the term validity for studies assessing the clinical accuracy of these decision-making tools with the treatment preference chosen by the surgeon, this paper addresses all validity studies as agreement studies.

## Materials & Methods

### Protocol

This systematic literature review was conducted according to the Preferred Reporting Items for Systematic Reviews and Meta-Analyses (PRISMA) guidelines.^11^ This study was not registered in the PROSPERO database. All steps of the review process were undertaken independently by two reviewers (KP and DS) and controversies resolved by a third reviewer (EK).

### Information Sources & Search Strategy

**Figure 1.**
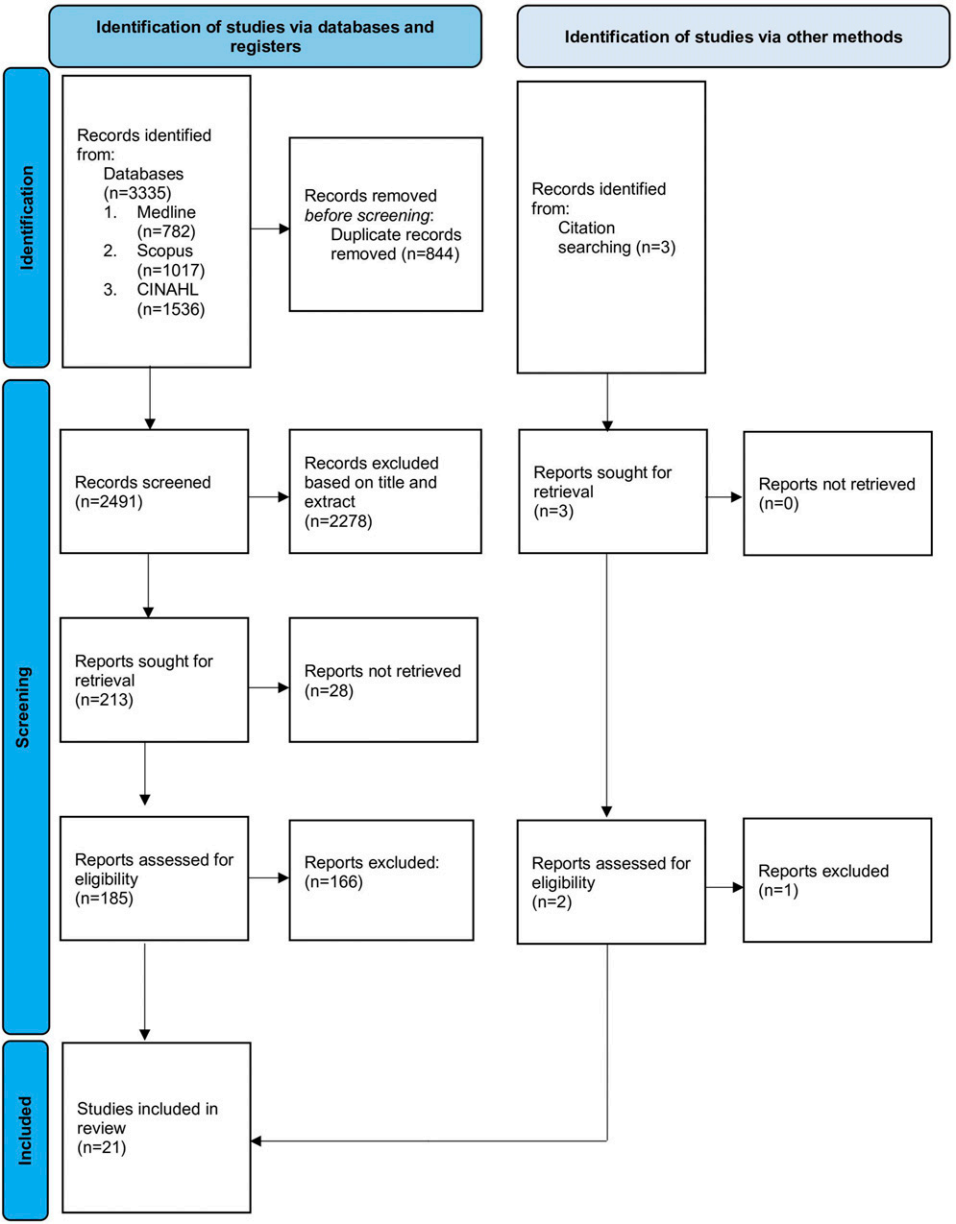
Prisma 2020 Flow Diagram.

Studies were acquired in April 2023 from three electronic databases: Medline, CINAHL and Scopus. Search terms were included based on relevant morphology, fracture type, and decision support tools. The complete search strategy is available in Supplemental Table 1. The search was conducted with restrictions on date (≥ 2000) and language (English only). References of the included studies were screened for relevant articles not in the electronic search and manually added (Figure 1).

### Eligibility Criteria

Two independent reviewers (KP and DS) performed abstract and subsequent full text screening using the following inclusion criteria:

1. Patients ≥18 years old with traumatic thoracolumbar fractures (+/− spinal cord injury)
2. Studies evaluating interobserver or intraobserver reliability (kappa score) of TLICS, AOSpine, or TL AOSIS
3. Studies evaluating agreement (validity) of TLICS or TL AOSIS
4. Studies evaluating the impact of imaging modality on TLICS, AOSpine, or TL AOSIS

Studies were excluded on the basis of:

1. Patients with pathological fractures (e.g. osteoporotic)
2. Patients with cervical or sacral fractures
3. Studies with duplicate cohort data

### Selection Process

Eligible papers were imported into Endnote reference management software and duplicates removed. Articles were then uploaded to Rayyan for title and abstract screening, where each was independently sorted by two reviewers (KP and DS) into inclusion or exclusion categories. Any discrepancies were resolved by a third reviewer (EK). The relevant full-text articles were then obtained and reviewed for inclusion by the two reviewers (KP and DS). Disagreements were resolved by discussion.

### Data Collection Process

Two researchers (KP and DS) worked independently to extract and document data from the included studies into tables. Relevant missing information was treated as meeting exclusion criteria. All included studies had the following data extracted: number of patients/cases and observers, the classification/scoring system assessed (TLICS, AOSpine, TL AOSIS) and purpose of the study (reliability, agreement, or imaging influence).

For reliability studies, kappa values were extracted for interobserver and intraobserver results. For agreement studies, the concordance between the tool vs the clinician’s treatment plan was recorded as a percentage value. For studies regarding influence of imaging modality on treatment decision making, percent treatment change based on CT +/− MRI was documented.

### Methodological Quality & Risk of Bias Assessment

Quality assessment of the included studies was conducted by two reviewers (KP and DS) using the modified COSMIN checklist adapted by Abedi et al, 2019^12^ for ordinal scoring systems. Overall score was decided in accordance with the “worst-score-counts” method.^13^ Any disagreements were resolved by consensus.

## Results

### Study Selection

The literature search identified 3335 results, of which 213 were chosen for full text analysis. 21 studies were included in the final review (Figure 1). All stages of screening were done independently by two researchers (KP and DS) to minimise bias. Conflicts were resolved by a third researcher (EK).

Four studies met the inclusion criteria but were excluded due duplicate cohort analysis. Joaquim et al, 2014^14^ was excluded due to having the same cohort as Joaquim et al., 2013.^15^ Sadiqi et al, 2015^16^ was excluded as it had analysis of the same cohort reported by Kepler et al., 2016.^17^ Rajasekaran et al, 2017^18^ was excluded as it had analysis of the same cohort as Rajasekaran et al., 2017.^9^ Whang et al, 2007^19^ was excluded as it had subgroup analysis of the cohort studied by Patel et al., 2007.^20^

### Study Characteristics

This study reviewed data from 21 papers, with a heterogenous quantity of patient and observers (Table 1). Papers were divided into three subcategories: reliability studies (n = 11), agreement studies (n = 6), and imaging modality influence studies (n = 4). Of the reliability studies, two papers also addressed agreement.^21,22^

**Table 1.** Study Characteristics.

	Number		Classification/Scoring System			Statistical Assessment			Modified COSMIN Quality Assessment
Author	Patients/Cases	Observers	TLICS	AOSpine	TL AOSIS	Reliability	Agreement	Imaging	
Chaves et al, 2015^22^	22	8	X			X	X		Fair
Cheng et al, 2017^25^	109	6		X		X			Fair
Kaul et al, 2017^28^	50	11	X	X		X			Fair
Kepler et al, 2016^17^	25	100		X		X			Fair
Lopes et al, 2018^27^	25	24		X		X			Good
Moore et al, 2014^21^	20	15	X			X			Fair
Patel et al, 2007^20^	25	21	X			X	X		Fair
Pishnamaz et al, 2018^29^	91	7	X	X		X			Fair
Reinhold et al, 2013^24^	129	5		X		X			Fair
Urrutia et al, 2015^30^	70	6		X		X			Fair
Vaccaro et al, 2013^26^	40	9		X		X			Fair
An et al, 2020^34^	110	5	X	X	X		X		Fair
Joaquim et al, 2013^15^	458	-	X				X		Good
Lambrechts et al, 2023^33^	815	-		X	X		X		Good
Nagi & Sakr 2022^35^	70	2	X	X	X		X		Good
Park et al, 2020^39^	328	-	X				X		Good
Santander & Rodríguez-Boto 2021^32^	458	2	X	X	X		X		Good
Alraddadi et al, 2024^38^	63	3		X				X	Good
Aly et al, 2022^37^	41	3		X				X	Good
Rajasekaran et al, 2017^9^	30	41		X				X	Fair
Winklhofer et al, 2013^36^	100	3	X					X	Good

Reliability studies explored the consistency of TLICS or AOSpine in classifying thoracolumbar fractures either by the same surgeon over a period of time (intraobserver) or by comparing different surgeons (interobserver). All included studies provided kappa scores and were interpreted with the Landis and Koch criteria.^23^ As these values are uniform, kappa was recorded as a value alone in the results tables. Agreement studies addressed the accuracy of TLICS or TL AOSIS matching the treatment recommended/provided by the clinician to patients with thoracolumbar fractures. Results were recorded as percentage. Papers regarding imaging modality influence on changing the TLICS (n = 1) or AOSpine (n = 3) classification and/or TL AOSIS explored the impact of CT +/− MRI in accurately assessing thoracolumbar fractures.

### Methodological Quality & Risk of Bias Assessment

Using the modified COSMIN checklist,^12^ included studies ranged from good to fair (Table 1). Overall quality was most significantly impacted by a paucity of weighted kappa used in statistical analysis. Due to limitation in available studies, no RCTs were included in this review.

Interobserver reliability kappa scores for AOSpine fracture type (A/B/C) were consistently lowest for type B (distraction injuries) amongst the eight included studies. This trend was present across all papers, regardless of patient number or observer characteristics. Collectively the highest reliability results were reported by Reinhold et al, 2013,^24^ with almost perfect agreement for A and C type fractures and substantial agreement for type B. In contrast, Cheng et al, 2017^25^ had the lowest interobserver reliability for all fracture types out of the eight included studies.

Intraobserver reliability for AOSpine kappa scores was reported as fracture type (A/B/C) and subtype (A0-A4/B1-B3/C). Regarding fracture type, three studies had almost perfect agreement,^17,26,27^ three studies had substantial agreement,^28-30^ and one study had moderate agreement.^25^ Kappa scores for fracture subtype were consistently lower than fracture type, with nil studies having almost perfect agreement. Substantial to moderate agreement was reported across the five studies that assessed subtype reproducibility, with B subtype fractures on average being less reliable than type A subtype.^17,26^

Five studies assessed ‘final score’ interobserver reliability for TLICS, with all studies including MRI images except for Pishnamaz et al, 2018.^29^ The average result was fair agreement, with the highest kappa value of 0.60 reported by Chaves et al, 2015^22^ vs the lowest of 0.23 by Pishnamaz et al, 2018.^29^ PLC interobserver kappa scores were reported by Kaul et al, 2017,^28^ Moore et al, 2014^21^ and Patel et al, 2007.^20^ Fair to moderate agreement was reported across the three studies, with all methods including MRI imaging. These studies also reported on morphology interobserver reliability with substantial variability in kappa scores. Moore et al, 2014^21^ had the lowest values for PLC and morphology and chose to focus their assessment purely on lower lumbar fractures (LLFs) (L3-L5).

Two studies measured intraobserver reliability for TLICS. Both Moore et al, 2014^21^ and Pishnamaz et al, 2018^29^ had very similar intraobserver kappa scores, despite significant timeline differences (six weeks vs three months). Compared to interobserver reliability, Pishnamaz et al, 2018^29^ found that intraobserver kappa for final score was higher (0.41 vs 0.23). This was also the case for Kaul et al, 2017,^28^ where interobserver kappa was 0.29 compared to the intraobserver kappa of 0.44 for TLICS ‘final score’.

TLICS and TL AOSIS were assessed for agreement (%) with conservative and/or surgical treatment. TLICS demonstrated consistently excellent accuracy for predicting conservative treatment, regardless of patient number. In contrast, there was substantially worse agreement for surgical treatment. This finding was strongly correlated to the surgeon’s preference to treat stable burst fractures surgically, with TLICS classifying these fractures as a score of 2 and therefore recommending conservative treatment.^15,31,32^ Lambrechts et al, 2023^33^ investigated the agreement of TL AOSIS. They found excellent concordance for both conservative and surgical treatment decision making. They further divided their assessment by scoring ranges: 0-3, 4-5, 6+ and found TL AOSIS had 100% success in predicting treatment for the ‘grey area’ of the scoring tool. Three studies directly compared TLICS to TL AOSIS.^32,34,35^ TL AOSIS was either equivalent or superior to TLICS for conservative treatment agreement and always superior for surgical treatment.

One study assessed the impact of imaging modality on classification and score for TLICS.^36^ The addition of MRI changed PLC status to injured from 18% (CT alone) to 42% of patients (CT + MRI).^36^ This contributed to a 33% change in classification and shifted treatment from conservative to surgical in 24% of patients.^36^

Three studies^9,37,38^ investigated the influence of CT and MRI on AOSpine classification and score. All studies found CT alone was sufficient. Higher sensitivity for B2 subtype fractures with the addition of MRI to CT imaging was reported by Rajasekaran et al., 2017.^9^ Aside from this finding, they reported CT was adequate for thoracolumbar assessment using AOSpine for all other fracture subtypes and did not change operative treatment recommendation rates.^9^ Aly et al, 2022^37^ specifically tested for low lumbar fractures (LLFs) (L3-L5). Their results supported that CT alone was sufficient to correctly classify LLFs and MRI was not indicated. In contrast Alraddadi et al., 2024^38^ assessed purely thoracic spine fractures (T1-T10) and found MRI changed classification in 16% of their cases with a 13% increase in surgical scoring. Although MRI aided in PLC injury assessment, they found ≥2 findings on CT was equivalent as a substitute.^38^

## Discussion

The overall objective of this systematic literature review was to address whether TLICS or AOSpine/TL AOSIS is best used in clinical practice through analysis of reliability, agreement, and imaging modality influence results of the twenty-one included studies.

### “Is TLICS or AOSpine Superior in Reliability and Agreement?”

#### Reliability

Interobserver reliability results for AOSpine were reported in seven of the included studies (Table 2). Of the fracture types (A/B/C), type B (tension-band) injuries were always the least reliable. This is likely due to difficulty in assessing the posterior tension band (equivalent to the posterior ligamentous complex in TLICS), a historically contentious part of the thoracolumbar spine.^25^ Reinhold et al, 2013^24^ had the highest kappa scores for fracture morphology types collectively, reporting almost perfect agreement for A and C types and substantial agreement for type B (Table 2). The authors of this study created the AOSpine classification and there is highly probable correlation between their familiarity with the tool and the results of this study. In contrast, Cheng et al, 2017^25^ reported the lowest scores for fracture type and interpreted this to be the result of the relatively inexperienced orthopaedic surgeons participating in the study (Table 2).

**Table 2.** Interobserver & Intraobserver Results of Reliability Studies (AOSpine).

Author	Patients/Cases	Imaging Used	Observers	Component Assessed	Inter-observer Reliability (κ)	Duration Between Assessments	Intra-observer Reliability (κ)
Cheng et al, 2017^25^	109 patients	XR + CT	6 orthopaedic surgeons	Fracture type	A: 0.385 B: 0.292 C: 0.552	1 month	A: 0.44 B: 0.49 C: 0.41
Reinhold et al, 2013^24^	110 cases	Not stated	5 spine trauma surgeons	Fracture type	A: 0.81 B: 0.71 C: 0.81	*Not assessed*	*Not assessed*
Kaul et al, 2017^28^	50 patients	XR + CT + MRI	11 attending spine surgeons (10 orthopaedic, 1 neurosurgeon)	Fracture type	A: 0.64 B: 0.40 C: 0.71	6 weeks	0.68 *(Combined)*
				Fracture subtype	*Not assessed*		0.61 *(Combined)*
Kepler et al, 2016^17^	25 cases	CT	100 spinal surgeons (naïve to AOSpine)	Fracture type	A: 0.80 B: 0.68 C: 0.72	1 month	0.85 *(Combined)*
				Fracture subtype	*Not assessed*		A: 0.57 B: 0.43
Pishnamaz et al, 2018^29^	91 patients	XR + CT	7 (board certified spine surgeons)	Fracture type	0.61 *(Combined)*	3 months	0.71 *(Combined)*
				Fracture subtype	*Not assessed*		0.57 *(Combined)*
Urrutia et al, 2015^30^	70 patients	XR + CT	6 (3 spine surgeons, 4 orthopaedic surgery residents)	Fracture type	A: 0.61 B: 0.57 C: 0.69	6 weeks	0.77 *(Combined)*
				Fracture subtype	*Not assessed*		0.71 *(Combined)*
Vaccaro et al, 2013^26^	40 cases	Not stated	9 fellowship-trained spine surgeons	Fracture type	A: 0.72 B: 0.58 C: 0.70	1 month	0.85 *(Combined)*
				Fracture subtype	*Not assessed*		A: 0.72 B: 0.43
Lopes et al, 2018^27^	25 cases	XR	24 (Group 1: 6 spine surgeons, Group 2: 18 orthopaedic residents)	Fracture type	A: 0.88 B: 0.76 C: 0.80	1 month	G1: 0.95 *(Combined)* G2: 0.82 *(Combined)*
				Fracture subtype	*Not assessed*		G1 A: 0.96 G1 B: 0.94 G1 C: 0.89 G2 A: 0.86 G2 B: 0.84 G2 C: 0.78

Seven studies reported on intraobserver reliability for AOSpine. There was substantial difference in the duration of time between assessments, ranging from one month to three months (Table 2). However, this variability did not seem to influence the results. Fracture type was on average more reliable than fracture subtype, with the greatest disparity reported by Kepler et al., 2016.^17^ Whether their findings were correlated to a relatively small number of cases (n = 25) or having the largest group of observers (n = 100) is unclear (Table 2). Overall, this trend suggests that it is easier to assess fracture type than subtype even with implied additional experience gained over time.

Interobserver reliability results for TLICS were reported in five of the included studies (Table 3). The average kappa values for ‘final score’ were rated as fair agreement, with Pishnamaz et al, 2018^29^ having the lowest score of 0.23. This finding may be attributed the authors using only X-ray and CT scans,^29^ opposed to the recommended MRI for PLC integrity assessment. Comparatively, Chaves et al, 2015^22^ had the highest kappa value of 0.60 and MRI scans were included for the majority of patient cases. Moore et al, 2014^21^ had the lowest values for PLC and morphology and chose to focus their assessment on lower lumbar fractures (L3-L5) only. The authors concluded that TLICS neglects to appropriately account for focal kyphosis in this region and its influence on a surgeon’s willingness to choose operative management.

**Table 3.** Interobserver & Intraobserver Results of Reliability Studies (TLICS).

Author	Patients	Imaging Used	Observers	Component Assessed	Inter-observer Reliability (κ)	Duration Between Assessments	Intra-observer Reliability (κ)
Kaul et al, 2017^28^	50	XR + CT + MRI	11 attending spine surgeons (10 orthopaedic, 1 neurosurgeon)	Final score	0.29	6 weeks	0.44
				Morphology	0.43		0.59
				PLC	0.47		0.55
Pishnamaz et al, 2018^29^	91	XR + CT	7 (board certified spine surgeons)	Final score	0.23	3 months	0.41
				Morphology	-		0.51
				PLC	-		0.47
Chaves et al, 2015^22^	22	XR + CT, MRI (not for all patients)	8 spine surgeons	Final score	0.60	*Not assessed*	-
				Morphology	-		-
Patel et al, 2007^20^	71	XR + CT + MRI	21 (orthopaedic and neurosurgery attendings, spine surgery fellows, senior and junior level resident physicians)	Final score	0.46	*Not assessed*	-
				Morphology	0.63		-
				PLC	0.45		-
Moore et al, 2014^21^	20 (low lumbar only)	CT + MRI	15 (6 orthopaedic spine surgeons, 4 neurosurgery attendings, 5 neurosurgery spine fellows)	Final score	0.25	*Not assessed*	-
				Morphology	0.39		-
				PLC	0.33		-

Only two studies compared interobserver to intraobserver reliability for TLICS (Table 3). Both Pishnamaz et al, 2018^29^ and Kaul et al, 2017^28^ reported moderate agreement for all components, despite significant variability in patient number, observers and duration between assessments (Table 3). When comparing ‘final score’, both studies had higher kappa scores for intraobserver reliability vs interobserver (Table 3). This likely reflects an improvement in the reliability of TLICS when there is more experience with the tool.

Comparing reliability, it is important to highlight that the included AOSpine studies assessed fracture morphology (type/subtype) vs TLICS studies which reported on final score, morphology, and PLC. Comparing morphology kappa results to fracture type, interobserver reliability was on average superior using AOSpine than TLICS across the included studies. This was also evident for intraobserver kappa results.

Two studies included in the review directly compared both tools using the same patient cohort.^28,29^ In the study by Kaul et al, 2017,^28^ they reported higher reliability kappa scores for interobserver and intraobserver AOSpine fracture type compared to TLICS morphology kappa scores. Their data set included the use of XR, CT and MRI. Comparatively, Pishnamaz et al, 2018^29^ only used XR and CT and reported a similar trend for intraobserver reliability between the two tools.

#### Agreement

Of the eight agreement studies analysed, three directly compared TLICS to TL AOSIS.^32,34,35^ These studies collectively found TL AOSIS was superior to TLICS when recommending surgical treatment. This finding is due to TL AOSIS more accurately differentiating between stable vs unstable burst fractures, improving surgical treatment agreement between TL AOSIS and the clinician. Santander & Rodríguez-Boto 2021^32^ reported significantly lower rates of surgical agreement than the other included studies (Table 4). For TLICS, this was due to classifying burst fracture patients as conservative (score of 2) when they were treated surgically. For TL AOSIS, neurologically intact A3 fractures are recommended to be treated conservatively. Subtype A4 earns a score of 5 and falls under the grey zone, recommending either conservative or surgical treatment at the surgeon’s discretion. The institution that ran the study performs surgery for both A3 and A4 subtypes to avoid possibility of progressive kyphotic deformity, influencing their results.^32^ Four studies analysed TLICS alone. Of these, Joaquim et al, 2013^15^ had the greatest disparity between conservative and surgical agreement (99.1% vs 46.6% respectively). They reported discordant patients had stable burst fractures (TLICS score of 2) that were treated surgically due to clinician preference to avoid possible future instability,^15^ a similar trend to Santander & Rodríguez-Boto 2021.^32^

**Table 4.** Results of Agreement Studies.

Author	Patients	System	% Agreement	Interpretation
Joaquim et al, 2013^15^	458	TLICS	Conservative: 99.1% (≤4)	TLICS was either too conservative or the authors overly aggressive with surgery. All discordant patients had stable burst fractures (TLICS score of 2) that were treated surgically.
			Surgical: 46.6% (≥5)	
Park et al, 2020^39^	328	TLICS	Conservative: 94.9% (≤4)	Mismatched patients with TLICS score of 2 that underwent surgery had stable burst fractures without neurological deficit.
			Surgical: 84.2% (≥4 score)	
Patel et al, 2007^20^	25	TLICS	95.4% (C*ombined*)	TLICS demonstrated excellent agreement.
Chaves et al, 2015^22^	22	TLICS	95.5% *(Combined)*	TLICS demonstrated excellent agreement.
Lambrechts et al, 2023^33^	815	TL AOSIS	Conservative: 96.1%	TL AOSIS demonstrated excellent agreement in conservative and surgical treatment decision making.
			Surgical: 97.5%	
			99.0% (0-3), 100% (4-5), 86.6% (6+)	
Santander & Rodríguez-Boto 2021^32^	458	TLICS	Conservative: 98.1%	Both TLICS and TL AOSIS demonstrated excellent agreement for conservative management.
			Surgical: 29.9%	
		TL AOSIS	Conservative: 98.1%	
			Surgical: 42.8%	
Nagi & Sakr 2022^35^	70	TLICS	85.7% (C*ombined*)	Both TLICS and TL AOSIS had similar agreement in conservative and surgical treatment recommendation.
		TL AOSIS	88.6% (C*ombined*)	
An et al, 2020^34^	110	TLICS	87.3% (C*ombined*)	TL AOSIS had superior agreement compared to TLICS, particularly with complete burst fractures.
		TL AOSIS	98.2% (C*ombined*)	

### “How Does Imaging Modality Influence TLICS and AOSpine Classification and Score?”

Four studies were included in this systematic review regarding the influence of imaging modality on classification and score of TLICS and AOSpine/TL AOSIS (Table 5). For TLICS, Winklhofer et al., 2013^36^ reported a 24% increase in conservative to surgical scoring (<5 to ≥5) when MRI scans were added after initial assessment with CT alone. This was attributed to improved accuracy in identifying PLC injury, increasing from 18% to 42% in the 100 patients evaluated.^36^ This finding is in keeping with the current literature, where MRI is considered an essential diagnostic tool for PLC injury and thus crucial to TLICS.^39,40^

**Table 5.** Results of Imaging Modality Influence Studies.

Author	Patients	Observers	System	Initial	Additional		Impact on Classification and Score with Additional Imaging	Authors Interpretation
Winklhofer et al, 2013^36^	100 consecutive patients	3 radiologists	TLICS	CT	MRI	Classification:	33% classification change with CT + MRI	MRI essential imaging modality for TLICS.
						Score:	24% increase in shift from conservative to surgical indication (score <5 to score ≥5)	
Rajasekaran et al, 2017^9^	30 fractures	41 spine surgeons	AOSpine/ TL AOSIS	XR	CT, then MRI	Classification:	XR alone (43%) -> +CT (62%) -> +MRI (64%) improvement in classification accuracy	MRI equivalent to CT except for B2 fracture subtype (higher sensitivity). CT sufficient for AOSpine. Type C only required XR.
						Score:	Assessment for need of surgery did not change after MRI	
Aly et al, 2022^37^	41 consecutive patients (L3-L5)	3 (2 radiologists, 1 spinal surgeon)	AOSpine/ TL AOSIS	CT	MRI	Classification:	4.9% classification change with CT + MRI (Type A- > Type B and vice-versa)	MRI unnecessary for low lumbar fractures, CT sufficient.
						Score:	MRI had nil influence on conservative vs surgical score recommendation	
Alraddadi et al, 2024^38^	63 consecutive patients (T1-T10)	3 (neuroradiologist, general radiologist, senior spinal surgeon)	AOSpine/ TL AOSIS	CT	MRI	Classification:	16% classification change from Type A to Type B with +MRI	CT sufficient for thoracic fractures. MRI beneficial for PLC but CT ≥2 findings sufficient for PLC integrity assessment.
						Score:	13% increase in conservative/surgical to surgical score (≤5 to ≥6)	

As MRI is not considered essential to AOSpine, it was important to clarify its impact on treatment agreement. All three studies assessing CT vs MRI influence on AOSpine/TL AOSIS concluded CT was sufficient as an imaging modality.^9,37,38^ When assessing LLFs, Aly et al, 2022^37^ had minimal influence from adding MRI to classification and score. Conversely Alraddadi et al., 2024^38^ isolated for purely thoracic fractures and had a more significant finding of 13% increase in surgical indication after MRI was added. However they concluded CT was viable as a lone imaging modality when greater than 2 findings for PLC damage were found, nullifying the benefit of MRI.^38^ This conclusion is supported by Barcelos et al, 2016,^41^ who found CT scans as a lone diagnostic tool were suitable at picking up PLC injury on average in 91.4% of type B or C thoracolumbar injuries.

## Limitations

This systematic review was restricted by a paucity of high-quality studies meeting inclusion criteria, highlighting the need for more prospective studies with larger patient cohorts to reduce bias. The generalisability of the findings is constrained by the significant heterogeneity in patient numbers, observers, and study methodologies. In addition, only one study included weighted kappa^27^ but did not report on the weighted scheme used. All other reliability studies reported unweighted kappa results which fails to account for the severity of disagreement, e.g. if a rating is misclassified as surgical when it should have been conservative, vs two values which are still are in conservative range. Weighted kappa is therefore more appropriate for systems like TLICs and AOSpine/TL AOSIS which are ordinal.^42^

Based on the conclusions reached, we recommend AOSpine/TL AOSIS be used in clinical practice. Future research should be focused on large, multi-centre prospective trials to build upon findings from previous retrospective studies.

## Supplemental Material

Supplemental Material - Which is the Superior Thoracolumbar Injury Classification Tool? TLICS Versus AOSpine 2013: A Systematic Review Supplemental Material for Which is the Superior Thoracolumbar Injury Classification Tool? TLICS Versus AOSpine 2013: A Systematic Review by Kristina T. Pidd, David Sadauskas, Vanesa Tomatis, and Ema J. Knight in Global Spine Journal
