# Supplementary material for: Which is the Superior Thoracolumbar Injury Classification Tool? TLICS Versus AOSpine 2013: A Systematic Review
Source: Global Spine J. 2024 Dec 25;15(4):2536–46. doi: 10.1177/21925682241311303 (PMC11670229; doi:10.1177/21925682241311303)
Supplement: Supplemental Material - Which is the Superior Thoracolumbar Injury Classification Tool? TLICS Versus AOSpine 2013: A Systematic Review [file sj-pdf-1-gsj-10.1177_21925682241311303.pdf]

Supplemental Data Document

Supplemental Table 1: Search Term Strategy

#	Medline	Scopus	CINAHL
1	spine/ or intervertebral disc/ or lumbar vertebrae/or thoracic vertebrae/ or vertebral body/ or thoracolumbar spine/	NA	Title: spine/ OR intervertebral disc/ OR lumbar vertebrae/ OR thoracic vertebrae/ OR vertebral body/ OR thoracolumbar spine/
2	(spin* or "intervertebral disc" or "lumbar vertebra*" or "thoracic vertebra*" or "vertebral body" or "thoracolumbar spin*").tw,kf.	(spin* OR "intervertebral disc" OR "lumbar vertebra*" OR "thoracic vertebra*" OR "vertebral body" OR "thoracolumbar spin*")	AB: (spin* OR "intervertebral disc" OR "lumbar vertebra*" OR "thoracic vertebra*" OR "vertebral body" OR "thoracolumbar spin*")
3	1 or 2	NA	
4	Spinal Fractures/	NA	TI Spinal Fractures/
5	(traum* or burst or "spin* fracture" or fractur*).tw,kf.	(traum* OR burst OR "spin* fracture" OR fractur*)	AB ((traum* OR burst OR "spin* fracture" OR fractur*))
6	4 or 5	NA	
7	Decision Support Techniques/	NA	TI Decision Support Techniques/
8	(decision adj1 (making guide or tool or making tool or support guide or support)).tw,kf.	(decision W/1 making W/1 guide OR tool OR "making tool" OR "support guide" OR support)	AB ((decision W/1 making W/1 guide OR tool OR "making tool" OR "support guide" OR support)
9	("decision support techniques" or "treat* guide" or "TLICS" or "Thoracolumbar Injury Classification" or "classification system" or "AOSIS" or "SIS" or "Load sharing classification" or TLISS or Magerl).tw,kf.	("decision support techniques" OR "treat* guide" OR "TLICS" OR "Thoracolumbar Injury Classification" OR "classification system" OR "AOSIS" OR "SIS" OR "Load sharing classification" OR TLISS or Magerl)	AB (("decision support techniques" OR "treat* guide" OR "TLICS" OR "Thoracolumbar Injury Classification" OR "classification system" OR "AOSIS" OR "SIS" OR "Load sharing classification" OR TLISS or Magerl)
10	7 or 8 or 9	NA	
11	3 and 6 and 10	NA	
